# Comparative Evaluation of a Commercial Herbal Extract and 0.2% Chlorhexidine Mouthwash on Three Periodontal Facultative Anaerobes: An In Vitro Study

**DOI:** 10.1155/2022/6359841

**Published:** 2022-12-30

**Authors:** Mohammad Mahdi Yaghooti Khorasani, Yasaman Mohammadi Kamalabadi, Somaye Salari Sedigh, Mina Jafari, Mostafa Sadeghi, Sahar Assar

**Affiliations:** ^1^Department of Endodontics, School of Dentistry, Rafsanjan University of Medical Sciences, Rafsanjan, Iran; ^2^University of Western Ontario, Department of Epidemiology and Biostatistics, London, Canada; ^3^Department of Periodontics, School of Dentistry, Rafsanjan University of Medical Sciences, Rafsanjan, Iran; ^4^School of Dentistry, Rafsanjan University of Medical Sciences, Rafsanjan, Iran; ^5^Department of Operative Dentistry, School of Dentistry, Rafsanjan University of Medical Sciences, Rafsanjan, Iran; ^6^School of Dentistry, Shiraz University of Medical Sciences, Shiraz, Iran

## Abstract

**Background:**

The application of herbal and/or chemical antimicrobial mouthwashes in addition to the mechanical methods of bacteria removal helps reduce the periopathogens and thus increase the periodontal tissues' health. The present study aimed to evaluate the antibacterial effect of Thymex (TMX) syrup on three periodontal facultative anaerobes in vitro and compare it with 0.2% chlorhexidine (CHX) mouthwash.

**Methods:**

In this in vitro experiment, the disc diffusion method was used to measure the inhibitory halo diameter (IhD) of *Enterobacter cloacae*, *Actinomyces viscosus*, and *Eikenella corrodens*. The paper discs containing TMX and CHX were placed on Mueller–Hinton agar media and cultured with the mentioned bacteria. Moreover, a blank disc containing distilled water was used as a control. From each of the three bacterial species, five samples were taken, and after 18 hours of storage in the incubator, the IhDs were measured in millimeters. A one-way ANOVA test and an independent sample *t*-test were used to compare the mean differences of IhDs between groups. The significance level was considered to be 0.05.

**Results:**

The IhDs ranged between 6.2–8.8 mm and 12.3–34 mm for TMX and CHX, respectively. CHX showed a more inhibitory effect on all three species of bacteria compared to TMX mouthwash (*P* < 0.001).

**Conclusions:**

Despite the inhibitory effect of TMX on bacterial growth, CHX showed significantly more antibacterial activity than TMX against three studied bacterial species.

## 1. Introduction

In recent years, novel innovations (such as metagenomics and culturomics) have provided powerful diagnostic methods/tools to investigate bacteria in the oral cavity [1]. More than 770 bacterial species have been found in the oral cavity so far, some of which are specifically associated with periodontal diseases [2]. *Actinomyces viscosus*, *Enterobacter cloacae,* and *Eikenella corrodens* are three facultative anaerobe species that are involved in the destruction of periodontal tissues and thus periodontal diseases [3–5].

The purpose of periodontal treatments is to reduce tissue inflammation, pocket depth, and pathogenic bacteria counts. Brushing teeth, using dental floss, doing precise scaling and root planning through the use of magnification systems [6], and also applying various herbal and chemical antimicrobial types of mouthwashes are all among the periodontal treatment methods that decrease the number of periodontal pathogens and/or limit their destructive activities and help to preserve periodontal tissues [7].

Chlorhexidine gluconate (CHX), as the gold standard for microbial control, is approved by the Food and Drug Administration (FDA) and the American Dental Association (ADA) and it is the most effective chemical antibacterial mouthwash due to its extensive antimicrobial activity and low toxicity [8]. Moreover, in lower concentrations (such as 0.1%, 0.12%, and 0.2%), CHX inhibits bacterial growth and is commonly used as a mouthwash; while in higher concentrations (for example, 2% and 5%), it would have bactericidal effects and be applied as an endodontic irrigant [9–11].

On the other hand, herbal extracts attract great interest as a result of showing antibacterial and/or anti-inflammatory effects and, simultaneously, fewer side effects compared to chemical agents [12–14]. For instance, *Thymus vulgaris* L. essential oil is among the compounds that are used for therapeutic activities due to its antibiofilm, antimicrobial, and antifungal effects [15, 16]. In an investigation, it was revealed that *T. vulgaris* essential oil had significant antibiofilm and antiquorum sensing activity against several Gram-negative and Gram-positive multidrug-resistant bacteria [17]. Moreover, it was suggested that *T. vulgaris* L., cinnamon, oregano, and clove essential oils could be added to the toothpaste to enhance the inhibitory effect against periodontal pathogens [18]. Furthermore, *T. vulgaris* as well as *Hyptis spicigera* essential oil were shown to have antibacterial, antibiofilm and inhibitory effects against cariogenic bacteria [19].

Based on previous studies, the Iranian *T. vulgaris* L. essential oil is comprised of carvacrol, thymol, linalool, and o-cymene [20–22]. One of the standardized extracts of the Iranian *T. vulgaris* L. is called Thymex (TMX), a brand name that is available in the form of syrup, and it is extracted from the aerial parts of the plant. Considering the increased desire for herbal products with fewer side effects compared to the chemical alternatives, it is essential to investigate the antibacterial effects of *T. vulgaris* L. on periodontal bacteria. Therefore, the present study aimed to evaluate the antibacterial effects of TMX syrup on three periodontal facultative anaerobes, *A. viscosus*, *E. cloaca*, and *E. corrodens,* and compare it with 0.2% CHX mouthwash as the gold standard.

## 2. Materials and Methods

**Figure 1:**
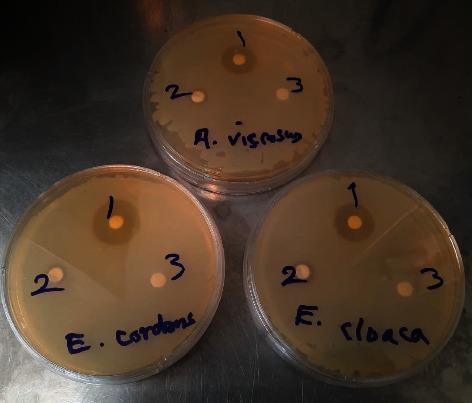
Sample plates containing *A. viscosus*, *E. corrodens*, and *E. cloaca* cultured on Mueller–Hinton agar. Paper discs impregnated with (1). chlorhexidine, (2). Thymex, and (3). distilled water (negative control).

In this in vitro study, two Gram-negative bacteria, *E. cloacae* (PTCC 1003) and *E. corrodens* (PTCC 1391), and one Gram-positive bacteria, *A. viscosus* (PTCC 1202), were selected and provided from the Persian Type Culture Collection (PTCC) [23]. The bacteria were first cultured in the liquid Brain Heart Infusion Broth (Merck KGaA, Darmstadt, Germany) at 37°C for 24 h, and then they were cultured on Trypticase Soy Agar (Merck KGaA) medium using the streak plate technique at 37°C for 48–72 h for the colonies to grow and become visible. Next, the single colonies (isolates) of the bacteria were removed, and based on the Kirby–Bauer disk diffusion method, the purified bacterial suspensions with a turbidity of 0.5 McFarland standard were prepared and used for the surface culture. To do so, a sterilized cotton swab was first dipped into the bacterial suspension; and then, by pressing the swab against the inner edge of the test tube, the excess liquid was removed from it. Then, the bacteria in the swabs were cultured on the surface of a plate containing Mueller–Hinton agar (Merck KGaA) using the spread plate method to inoculate the entire surface with the bacteria (Figure 1). The culture plates were left for 2–5 min so that their moisture could be absorbed. Paper discs (Padtan Teb Co., Tehran, Iran) impregnated with TMX (Iran Darouk, Tehran, Iran) and CHX (Behsa Co., Iran) were placed in an incubator to dry. They were then carefully put on the culture medium in the plates and gently pressed against the agar surface for the entire disk to be in contact with the agar. Blank discs (filter paper containing distilled water) were used as the negative control. Incubation was carried out at 37°C under normal atmospheric conditions for 24 h. Each of the studied bacterial species was cultured five times to increase the accuracy of the study [24]. All the cultured plates were included except those with insufficient bacterial growth (i.e., light cultures). The negative control discs (blank, without any antimicrobial material) were also used for all three bacterial species. The inhibitory halo diameter (IhD) was measured in millimeters using a caliper and recorded in the information forms [25]. In the end, 15 plates (with three discs on each plate) were cultured and studied. After carrying out the laboratory stages and giving each culture plate a number, the IhD for each plate was measured and recorded in the checklist.

### 2.1. Statistical Analysis

The data were analyzed using SPSS 21.0 (IBM SPSS Statistics for Windows, Version 21.0, NY: IBM Corp.). The IhD (in millimeters) was measured for each bacterial species as the mean ± SD (standard deviation). The Shapiro–Wilk test was used for normality assessment (*P* > 0.05). Levene's test was used to assess variance equality (*P* > 0.05). One-way ANOVA test and an independent sample *t*-test were conducted to compare the means between different groups of bacteria. The *P* value less than 0.05 (*P* < 0.05) was considered significant.

## 3. Results

**Figure 2:**
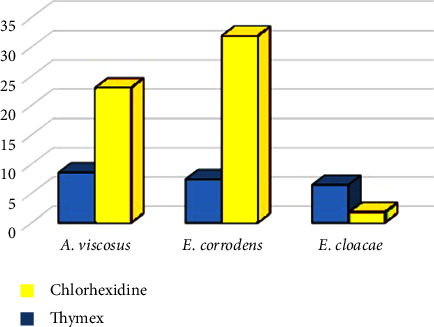
The difference between inhibitory halo diameters of the *E. corrodens*, *A. viscosus*, and *E. cloacae*.

The IhD of the three studied bacteria is represented in Table 1 and Figure 2. The IhDs ranged between 6.2-8.8 mm and 12.3–34 mm for TMX and CHX, respectively. The largest and smallest means of the IhD for TMX were 8.58 ± 0.16 mm and 6.46 ± 0.18 mm for *A. viscosus* and *E. cloacae,* respectively. Also, these amounts for CHX were 31.96 ± 1.27 mm (*E. corrodens*) and 13.04 ± 0.57 mm (*E. cloacae*), respectively.

As shown in Table 2, the difference in means of the IhD between the two groups of TMX and CHX was significant among all three studied bacteria (*P* < 0.001). In addition, the comparison of the IhD between CHX and the control group for all three studied bacteria showed significant differences (*P* < 0.001). However, the differences between TMX and the control group for *E. corrodens* and *A. viscosus* were not significant (*P*=0.998 and *P*=0.720, respectively).

## 4. Discussion

This in vitro study evaluated the antibacterial effects of TMX, a *T. vulgaris* L. extract, and compared it with 0.2% CHX mouthwash. TMX showed an inhibitory growth effect on all three studied bacteria species. However, the results revealed that CHX had a significantly greater antibacterial effect compared to TMX. Moreover, TMX showed the most antibacterial effect on *A. viscosus*, while CHX showed the most antibacterial effect on *E. corrodens*.

In this study, TMX inhibited the growth of the studied periodontal facultative anaerobes. Concordant with the current result, other studies also suggested that *T. vulgaris* L. extract had antibacterial effects [17, 18, 26, 27]. For instance, Abuidris et al. [28] reported that 2% CHX, 5.25% sodium hypochlorite, and 2% *T. vulgaris* L. essential oil exhibited the most efficient antibacterial effects against *Enterococcus faecalis,* respectively. Moreover, Dehghani et al. [26] reported that *T. vulgaris* L. essential oil at 200–400 ppm and ceftriaxone and tetracycline had similar antibiotic effects. In addition, some studies showed that *T. vulgaris* L. essential oil inhibited the growth of some species of multidrug-resistant bacteria [17, 27]. Also, due to the *T. vulgaris* L. essential oil's antibacterial effect, it was recommended that it should be added to fluoride-free toothpaste to reduce dental caries and periodontal diseases [18]. As reported in studies on *T. vulgaris*, many other herbal extracts and compositions, such as Calendula officinalis, neem, tulsi, pudina, clove oil, *ajwain*, *triphala*, and *baicalin* (a natural molecule found in Baical Skullcap, *Scutellaria baicalensis Georgi-*roots), were shown to have antibacterial, antiplaque, and anti-inflammatory effects as well [14, 29, 30], suggesting that herbal agents are useful to be included in oral health products.

The current study indicated that CHX had a more significant antibacterial effect than TMX. This is in accordance with the results of most of the studies comparing herbal mouth rinses with CHX [18, 30–32]. For instance, Pathan et al. [32] reported that the antibacterial effect of CHX was higher than that of herbal mouthwash. In another study [30], CHX was shown to inhibit plaque growth more than herbal mouthwash. Moreover, Carvalho et al. [18] showed that CHX had a stronger antibacterial effect than *T. vulgaris* L. essential oil, and these two substances synergistically inhibited the growth of *Streptococcus mutans*. Also, in another study comparing the use of CHX and *T. vulgaris* L. extract in children, it was reported that 30 minutes after using CHX mouthwash, the oral *S. mutans* count was decreased in comparison with *T. vulgaris* L.; however, one week after using these two types of mouthwash, the bacterial count was less increased in those children who had used *T. vulgaris* L. extract compared to the ones who had used CHX [31]. In contrast, Rezaei et al. [33] manifested that the herbal mouthwash containing ethanol extract of *Salvadora persica* and Aloe vera gel was significantly more influential in reducing gingival index in intubated patients in comparison with CHX mouthwash, suggesting herbal mouthwash has more antibacterial activity. Most of the studies revealed that CHX is more efficient in reducing oral bacterial activity. However, different compositions of herbal mouthwashes might show different efficiencies in antibacterial activity, and some of them might be comparable with CHX as the gold standard mouthwash. Also, previous research has suggested that herbal mouthwashes, due to their lesser side effects and better taste perception, could be good alternatives for CHX [34, 35].

This in vitro study compared the antibacterial effects of TMX syrup and CHX mouthwash on three specific periodontal facultative anaerobes outside the oral environment. Novel innovations such as metagenomics and culturomics in recent years have provided more precise diagnostic methods that result in finding a larger variety of periodontal pathogenic bacteria [1]. Moreover, it has been shown that with the help of magnification systems, more efficient mechanical bacteria and plaque removal can be obtained [6]. The application of herbal and/or chemical antimicrobial mouthwashes in addition to mechanical methods of bacteria removal helps increase in the health of periodontal tissues.. Further research is needed to evaluate the antibacterial activity of herbal mouthwashes such as TMX and compare them with CHX mouthwash on different bacterial species in vivo and also in clinical trials for patients with periodontal diseases.

## 5. Conclusions

In the present study, TMX showed an inhibitory effect on the growth of the studied periodontal pathogens. However, CHX revealed significantly greater antibacterial activity than TMX against all three bacterial species. Further studies should be conducted to examine the antibacterial effects of TMX in both in vivo studies and clinical trials and compare its efficacy with CHX.

## Figures and Tables

**Table 1:** The IhD difference among *E. corrodens*, *A. viscosus*, and *E. cloacae*.

Mouthwash	Bacteria	IhD (mm)			
		Minimum	Maximum	Mean ± SD^^^	*P* ^*∗*^
TMX	*E. corrodens*	7.1	7.7	7.42 ± 0.22	<0.001
	*A. viscosus*	8.4	8.8	8.58 ± 0.16	
	*E. cloacae*	6.2	6.7	6.46 ± 0.18	

CHX	*E. corrodens*	30.5	34	31.96 ± 1.27	<0.001
	*A. viscosus*	22.5	24.2	23.08 ± 0.66	
	*E. cloacae*	12.3	13.7	13.04 ± 0.57	

CHX, chlorhexidine; TMX, Thymex; IhD, inhibitory halo diameter; *∗*, one way ANOVA, *P* < 0.05 considered significant; ^, standard deviation.

**Table 2:** Effects of TMX and CHX on IhD of the three bacterial species.

Mouthwash	IhD (mm) mean ± SD^^^		*P* ^*∗*^
	TMX	CHX	
*Bacteria*:			
*E. corrodens*	7.42 ± 0.22	31.96 ± 1.27	<0.001
*A. viscosus*	8.58 ± 0.16	23.08 ± 0.66	<0.001
*E. cloacae*	6.46 ± 0.18	13.04 ± 0.57	<0.001

CHX, chlorhexidine; TMX, Thymex; IhD, inhibitory halo diameter; *∗*, independent sample *t*-test, *P* < 0.05 considered significant; ^, standard deviation.

## Data Availability

The datasets used and/or analyzed during the current study are available from the corresponding author upon reasonable request.
